# High Aspect Ratio Nanoscale Pores through BCP-Based Metal Oxide Masks and Advanced Dry Etching

**DOI:** 10.1021/acsami.3c09863

**Published:** 2023-10-20

**Authors:** Aislan Esmeraldo Paiva, Michael S. Gerlt, Nino F. Läubli, Nadezda Prochukhan, Jhonattan Frank Baez Vasquez, Gabriele S. Kaminski Schierle, Michael A. Morris

**Affiliations:** † AMBER Research Centre/School of Chemistry, Trinity College Dublin, Dublin D02 CP49, Ireland; ‡ Department of Biomedical Engineering, Lund University, Lund 22363, Sweden; § Department of Mechanical and Process Engineering, ETH Zürich, Zürich 8092, Switzerland; ∥ Department of Chemical Engineering and Biotechnology, University of Cambridge, Cambridge CB3 0AS, U.K.

**Keywords:** block copolymer, metal infiltration, DRIE, high aspect ratio, porous silicon

## Abstract

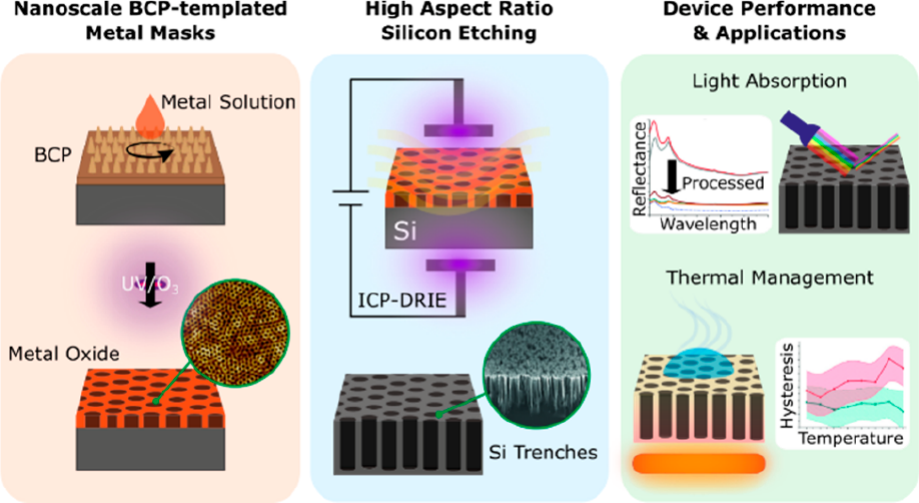

The reliable and regular modification of the surface properties of substrates plays a crucial role in material research and the development of functional surfaces. A key aspect of this is the development of the surface pores and topographies. These can confer specific advantages such as high surface area as well as specific functions such as hydrophobic properties. Here, we introduce a combination of nanoscale self-assembled block-copolymer-based metal oxide masks with optimized deep reactive ion etching (DRIE) of silicon to permit the fabrication of porous topographies with aspect ratios of up to 50. Following the evaluation of our procedure and involved parameters using various techniques, such as AFM or SEM, the suitability of our features for applications relying on high light absorption as well as efficient thermal management is explored and discussed in further detail.

## Introduction

The ability to control relevant surface properties, such as cooling and light absorption, at the nanoscale level is essential for a wide variety of applications and fields, including thermodynamics,^1,2^ energy development,^3,4^ and materials science.^5−7^ Evaporative cooling, also known as evaporative heat transfer, describes the natural process where a liquid changes its state to vapor, resulting in a local decrease in temperature. Hence, it plays a fundamental role in refrigeration and air conditioning systems,^8^ cooling towers for power plants,^9^ and thermal management of electronics.^10^ On the other hand, light absorption is the process by which a material absorbs light and converts it into other forms of energy, such as heat or electricity. It is, therefore, an essential mechanism in solar energy conversion devices,^11^ photovoltaic cells,^12^ and optical coatings.^13^

One effective approach to enhance the performance of evaporative cooling and light absorption lies in the development of strictly controlled porous structures. Porous materials, in contrast to planar geometries, offer a higher surface to volume ratio, which enables efficient heat and mass transfer^14^ as well as enhanced light absorption.^15^ To achieve high spatial resolution patterns with low and periodic arrangements, block copolymer (BCP) lithography is a promising technique as it produces porous substrates at the nanoscale with well-defined and tunable morphologies. However, block copolymer lithography has, so far, been limited to small topographical heights with low aspect ratios because of the poor etch selectivity of the BCP derived etch mask, made of a polymer block, over the substrate.^16^ This laboratory has been central in developing infiltration methods to yield hard masks that allow for larger aspect ratios,^17−19^ while other potential methods to create hard masks have been reported and involve sol–gel^20−22^ and sputtering approaches.^23,24^ Nevertheless, the generation of hydrophobicity, which enables effective evaporative cooling, or effective light scattering, at very high (>25) aspect ratios remained challenging. In addition, such very deep features have not been achieved to date and require advanced etching to prevent damage to the nm size arrangements during the etch process.

At the core of the BCP-based lithography is the controlled self-assembly of the engineered polymer, which is composed of two or more chemically distinct polymer blocks, into periodic nanostructures,^25^ which can act as templates.^26,27^ These nanostructures can then further be transferred into the underlying substrate using etching techniques once the BCP is converted into a mask,^28−30^ e.g., using advanced processing methods such as deep reactive ion etching (DRIE) suitable for the generation of high aspect ratio features with precisely controlled geometries.^31^ However, thus far, the number of successful attempts combining well-defined BCP masks with sufficiently high structural stability and reliable anisotropic etching at the nanoscale is limited, and, so far, none have been able to create very high aspect ratio pores at a substrate surface.

In this study, we present DRIE processing suitable for the fabrication of deep pore nanostructures with a high aspect ratio when combined with optimized BCP-templated etch masks. Two poly(2-vinylpyridine-*b*-styrene) (P2VP-*b*-PS) systems were employed for the mask production, with their phase separation induced by a solvent vapor annealing (SVA) method, leading to vertically aligned polystyrene (PS) cylinders with diameters of 32 and 343 nm. A wide range of feature sizes was selected to demonstrate the capability of hard mask preparation. Then, the porous masks were fabricated by selectively infiltrating a chromium precursor and subsequently oxidizing/removing the BCP template, resulting in uniform porous metal oxide patterns. The different chromium oxide masks were finally used to transfer the features into a bulk silicon substrate via a newly developed three-step DRIE process. While previous work^32^ demonstrated the use of BCP-based porous structures to be applied as a hard mask in simple dry etching processes, the current process goes significantly further via the optimization and adaptation of the mask, e.g., by fabricating different pore sizes, which is crucial to demonstrate enhanced versatility of the fabrication procedure. Furthermore, we substantially extend the processing and application capabilities through the combination of our BCP masks with a custom and advanced dry etching technique to enable the creation of trenches with unprecedented aspect ratios and evaluate the resulting features and related process parameters in further detail. Moreover, the development of very high aspect ratio features is unique, as it opens possibilities for through-substrate structures to enable molecular transport across well-defined membranes. The resulting pores and devices were tested for potential applications through reflectance and thermal contact angle (CA) measurements, with the results highlighting low reflectance values as well as low CA hysteresis, both of which are properties that are highly desirable for various fields, including thermal management and solar energy conversion.

## Experimental Methods

### Chemicals and Materials

Chemicals and materials were used as received. B-doped-type silicon (111) dummy wafers with a native oxide layer (≅2 nm; University Wafer) were used as substrates. Two poly(2-vinylpyridine-*b*-styrene) (P2VP-*b*-PS) BCP systems with their respective number-average molecular weight (MW) and polydispersity index (PDI) were obtained from Polymer Source Inc.: BCP1 (MW_P2VP_ = 60 kg mol^–1^, MW_PS_ = 26 kg mol^–1^, PDI = 1.15) and BCP2 (MW_P2VP_ = 598 kg mol^–1^, MW_PS_ = 189 kg mol^–1^, PDI = 1.22).

Acetone (99.0%), toluene (99.8%, anhydrous), tetrahydrofuran (99.8%, anhydrous), chloroform (99.9%, anhydrous), isopropanol (99.5%, anhydrous), 1-butanol (99.8%, anhydrous), and chromium(III) nitrate nonahydrate (≥99.99%) were sourced from Sigma-Aldrich. All of the procedures were conducted at room temperature, unless otherwise specified.

### Phase Separation of the P2VP-*b*-PS Films and Fabrication of the Hard Masks

Phase separated P2VP-*b*-PS films were prepared according to previous studies.^32,33^ Silicon wafers were cut into 4 cm^2^ squares and subjected to ultrasonic cleaning in acetone for 20 min, followed by drying with a stream of N_2_. Solutions of BCP1 (1.5 wt %) and BCP2 (2.0 wt %) were prepared in a 1:4 mixture of toluene and tetrahydrofuran. Each solution was spin-coated onto the Si substrates at 3000 rpm for 30 s using a vacuum-free Ossila spin coater. Subsequently, the coated substrates were placed in 150 mL jars, each containing a glass vial with 2 mL of chloroform. The jars were stored in a refrigerator at 5 ± 2 °C for 40 min (BCP1) or 96 h (BCP2). Finally, the samples were removed from the jars and allowed to dry at room temperature.

A solution of 2.0 wt % % chromium nitrate in butanol was prepared and applied to the phase-separated BCP thin films using spin coating at 3000 rpm for 30 s. The coated films were then exposed to a UV/ozone (UVO) treatment in a chamber with two low-pressure mercury lamps (with an output current ranging from 0.8 to 0.95 A, power between 65 and 100 W, and emissions at 184.9 and 253.7 nm) using a PSD Pro Series Digital UV Ozone System (Novascan Technologies, Inc.). The samples were placed 4 cm away from the UV source, and the process was conducted for 2 h. Following this, the substrates were heated in a furnace (Thermo Scientific FB1415M) to 400 °C for 1 h.

### Deep Reactive Ion Etching

Deep reactive ion etching (DRIE) was conducted on the hard-mask-covered substrates in a PlasmaPro Estrelas100 (Oxford Instruments) using C_4_F_8_ and SF_6_ gases. During the etching procedures, the wafers were maintained at a temperature of 0 °C by utilizing liquid nitrogen backside cooling. The used process parameters are listed in Table 1. For simplicity, the process was summarized in three steps. However, the actual recipe contained intermediate phases between the main steps that are applied to reduce the pressure of the chamber, leading to an increased mean free path length, or to an exchange of the gas environment, as well as to a split of the first step into two components and, by that, to a reduction in the duration of the pre-etching passivation (see SI Figure S2).

**Table 1:** DRIE Etching Process Parameters^a^

Steps	Duration [ms]	Pressure [mTorr]	ICP power [W]	HF power [W]	C_4_F_8_ flow [sscm]	SF_6_ flow [sscm]
Passivation (I)	1350	120	2500	0	280	10
Break through (II)	400	20	2000	75	10	200
Chemical and physical etching (III)	500	120	2500	50	10	800

a Each etch cycle consists of three steps. The main process parameters are the duration time of the individual steps, the pressure inside chamber, the inductively coupled plasma (ICP) power affecting plasma density, the HF power inducing the acceleration of the ions toward the target, and the gas flow.

### Characterization

Atomic force microscopy (AFM) topographical images of the samples were captured by using a Park XE-100 microscope (Park Systems). Noncontact mode was selected for the imaging process, utilizing a AC160TS cantilever (with a force constant of 26 N m^–1^ and resonance frequency of 300 kHz). The measurement of 100 features was used to calculate the pore diameter and pore–pore spacing, with the results being expressed as the mean ± standard deviation.

SEM micrographs were captured using a Zeiss Ultra Plus microscope with an accelerating voltage of 2 kV, a working distance of 4–5 mm, and a secondary electron (SE2) detector. The pore surface area was evaluated via ImageJ software. The measurement of 50 features was used to calculate the pore depth, with the results being expressed as mean ± standard deviation.

To evaluate the metal oxide layer thickness, composition, and structure, the substrate was sectioned using a Zeiss AURIGA focused ion beam (FIB), utilizing an ion beam current ranging from 4 nA to 50 pA and accelerating voltages of 30 and 15 kV. Subsequently, images obtained through scanning transmission electron microscopy (STEM) were combined with energy dispersive X-ray spectroscopy (EDX) data. This was done using a FEI Titan G2 80-300FEG S/TEM with a Schottky-type electron gun operated at 300 kV and a Bruker XFlash 6T-30 detector with a resolution of 129 eV.

Reflectance measurements were acquired on a LAMBDA 365 UV/vis Spectrophotometer (PerkinElmer) coupled with a 50 mm transmission-reflectance integration sphere (5 nm slit) in the range of 200–1100 nm.

A custom-designed system was utilized to measure the dynamic contact angle (CA) on 10 random regions of the samples. The room temperature and relative humidity on the day of the experiment were 20 ± 2 °C and 50 ± 10%, respectively. Experiments were conducted at atmospheric pressure and with a duration of 3–5 s.

A high-speed camera was employed to capture the advancing and receding CAs of water at a sampling rate of 60 Hz. The liquids were dispensed using a 35-gauge needle (with an outer diameter of 135 μm) at a flow rate of 5 nL·s^–1^, resulting in droplet volumes of 100 nL. Before measuring the CAs, a diluted oxalic acid solution (1% wt.)^34^ was used to remove any remaining Cr oxide mask. A heating PID-controlled stage was used to change the temperature of the experiment. The experimental results are displayed with error bars based on standard error or 95% confidence intervals (where stated).

## Results and Discussion

### BCPs Enable Porous Hard Masks with Precisely Controlled Dimensions

**Figure 1:**
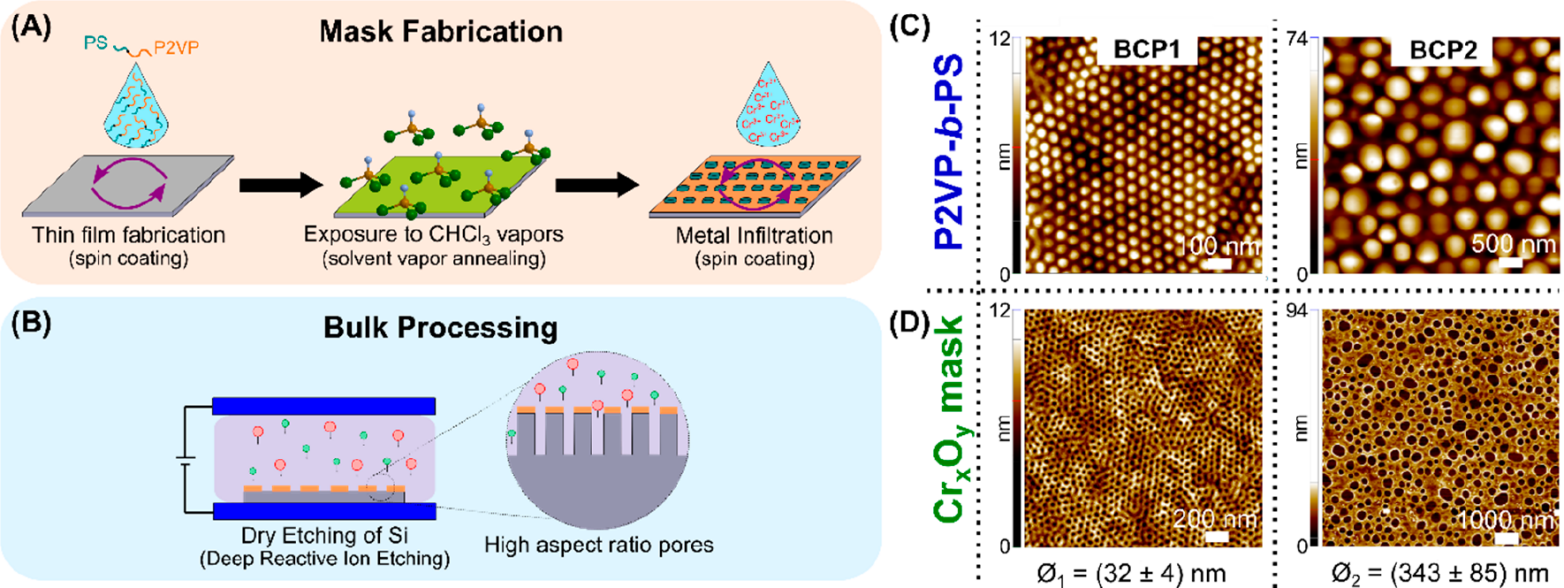
Fabrication procedure can be separated in two main components. (A) Schematic of the metal oxide porous layer mask fabrication and (B) subsequent bulk etching of Si. AFM images of (C) P2VP-*b*-PS ordered thin films: BCP1 (86 kg mol^–1^) and BCP2 (787 kg mol^–1^); and (D) chromium oxide hard masks with pore diameters (Ø) of 32 ± 4 and 343 ± 143 nm, respectively.

Despite increasing need in miniaturization, the fabrication of stable masks with nanoscale features suitable for etching processes remains challenging.^35^ BCPs possess the remarkable ability to phase separate, e.g., via solvent vapor annealing (SVA), to create vertically aligned structures which, through subsequent selective metal infiltration,^36^ permit the formation of metal oxide layers. Figure 1A,B display the general procedure for the fabrication and application of such porous hard masks on silicon (Si) substrates for subsequent bulk etching. Through the contact of the BCP thin film with chloroform vapors, i.e., a nonselective solvent, unfavorable interactions between the P2VP (poly 2-vinylpyridine) and the PS blocks can be reduced, which leads to the vertical alignment along the surface plane of the domains.^32^ Therefore, based on the two molecular weights studied in this work, different morphologies were obtained. Figure 1C presents topographical atomic force microscopy (AFM) images of the self-assembled polymeric thin films with the PS cylinders of varying dimensions, i.e., diameters of about 32 and 343 nm, being embedded within the P2VP matrix. The significant difference in the annealing time between the two BCPs (see Experimental Methods) is related to the chain entanglement, as longer chains require more energy or time to self-order.^37,38^

Following the formation of the polymeric thin film, the infiltration of the Cr ions and the subsequent oxidation of the BCP led to porous chromium oxide (Cr_*x*_O_*y*_) thin layers, previously confirmed by XPS and EDX,^32^ with a thickness of ∼10–25 nm, as shown in Figure 1D. To establish the optimum concentration of the chromium precursor, BCP1 thin films were infiltrated with different concentrations of chromium nitrate solutions, while 2.0% wt. was determined as the highest possible concentration without overfilling of the pores (SI Figure S1). The optimized metal oxide masks did not present any significant changes in the pore diameter when compared to the initial polymer and resulted in average pore diameters for BCP1 and BCP2 of 32 ± 4 and 343 ± 130 nm, respectively. The successful transfer of the BCP template layout and structure to the Cr oxide masks strongly suggests that the PS cylinders were vertically aligned throughout the P2VP matrix. Nevertheless, further investigations are needed to confirm their verticality.

### Combination with Dry Etching Permits Fabrication of Very Deep Porous Structures with High Aspect Ratios in Silicon Substrates

The pattern of the chromium oxide hard masks was transferred into the Si substrate via inductively coupled plasma deep reactive ion etching (ICP-DRIE). Figure 1B shows a sketch of the fabrication procedure, while SI Figure S2 introduces the process cycle in further detail. A standard two step Bosch process^39^ was expanded by an intermittent step to allow for higher controllability during the etching procedure and to permit the reliable fabrication of high aspect ratio structures with minimal variations in etch angles as necessary during the application of nanoscale masks.^40^ Accordingly, the custom-designed etch cycle consisted of the following three steps: (I) deposition of a C_4_F_8_ protective layer covering the bottom and the sidewalls of the pores, (II) removal of the protective layer on the bottom of the pores through primarily physical etching (anisotropic) based on accelerated ions using SF_6_, and (III) combined chemical (isotropic) and physical etching of the protective layer on the sidewalls and the exposed silicon substrate at the bottom of the pores with SF_6_. During the additional second step, in contrast to the standard Bosch procedure, the ions were accelerated toward the wafer through the application of a strong high-frequency (HF) electric field, leading to a highly anisotropic etching with low selectivity. Finally, while traditional processes use low HF power paired with high gas flows during the last step to improve selectivity and promote isotropic chemical etching of Si, thanks to the high stability of the chromium oxide mask and in contrast to previous work,^40^ our procedure permitted the use of continuously high HF powers in combination with a reduction in the duration of the third etch step. This, in turn, led to an increase in the proportion of the ongoing anisotropic silicon etching and, as such, minimized challenges associated with chemical etching and diffusion limitations within the long yet narrow pores.

**Figure 2:**
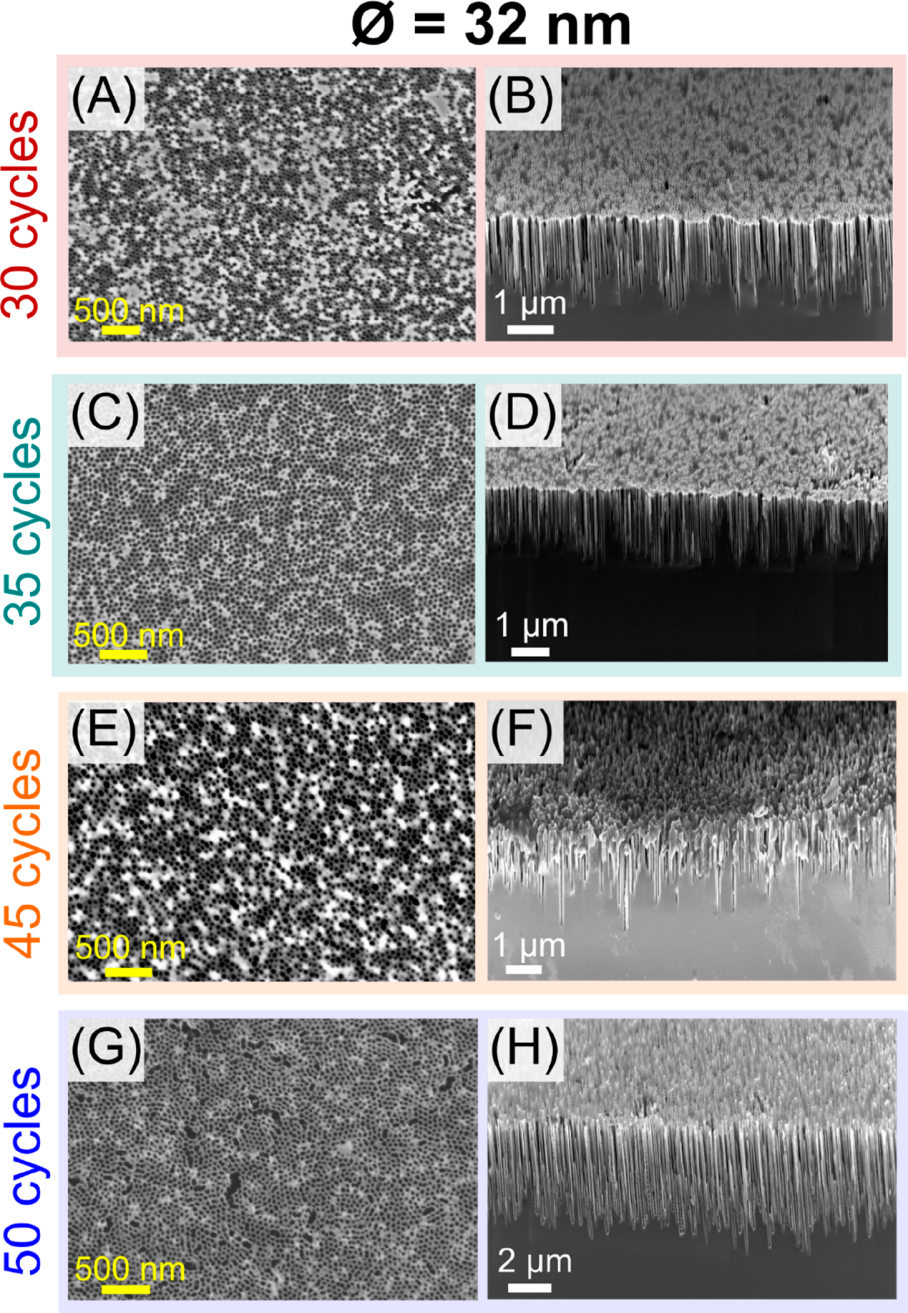
Above 35 etch cycles, the 32 nm mask and pores become unstable. The figures show top down and cross section scanning electron microscopy (SEM) micrographs of the etched silicon substrates with masks containing 32 nm pores and varying numbers of etch cycles: (A,B) 30 cycles, (C,D) 35 cycles, (E,F) 45 cycles, and (G,H) 50 cycles.

Based on the above process, increasing numbers of etch cycles were applied to extend the depth of the pores and to evaluate device performance and process reliability (Figure 2). Accordingly, for 32 nm pores, 30 cycles led to a depth of 1.32 ± 0.26 μm (Figure 2B), i.e., an aspect ratio of more than 40, while 35 cycles produced pores with a depth of 1.56 ± 0.21 μm and a high aspect ratio of about 50 (Figure 2D). The variation in depth is likely associated with minor variations in the diameter of each pore, which, due to limitations in gas exchange, result in faster or slower etching rates and, subsequently, lead to variations in pore depth also known as etch lag.^40,41^ Nevertheless, these exceptional results demonstrate that the stability of the chromium oxide mask was sufficient to, in combination with the optimized etching recipe, allow for the production of high aspect ratio features while preventing complete mask removal during the procedure. Further and to the best of the authors’ knowledge, the production of features with similarly high aspect ratios and comparable feature sizes using standard DRIE equipment has not yet been demonstrated, as highlighted in Table 2, which summarizes previous aspect ratios of nano to microscale structures.

**Table 2:** Etch Depths Reported as Achieved through Silicon Etching Processes at Similar Length Scales Achieved Only Lower Aspect Ratios.

Etch type	Aspect ratio	Feature size (nm)	Depth	Reference
Cryo-ICP (SF_6_/O_2_)	4.2	12	50 nm	(44)
ICP (HBr/O_2_)	5	8–9	45 nm	(45)
ICP (Cl_2_/SF_6_)	10	50	500 nm	(46)
ICP/RIE (SF_6_/C_4_F_8_)	17	75	1.3 μm	(47)
Cryo-RIE (SF_6_/O_2_)	26.5	400	10.6 μm	(48)
ICP/RIE (SF_6_/C_4_F_8_)	50	32	1.6 μm	This work

To study the morphology of the pores after etching, scanning transmission electron microscopy and energy dispersive X-ray spectroscopy (STEM-EDX) analyses were conducted (SI Figure S3). The results showed that our procedure was successful in preserving the crystalline structure of the silicon substrate, as demonstrated by the noticeable lattice spacing corresponding to the (111) plane of the Si FCC structure.^42^ As no amorphization was detected in the TEM observable area and due to the low processing temperature and short duration of the etching, it can also be assumed that no changes in the Si lattice structure are present in areas further away from the contact with the etching gases. Additionally, elemental mapping displayed the presence of the remaining Cr oxide mask as well as the other expected elements. However, from 35 etch cycles and onward, the pores started to lose their structure and stability, as presented in Figure 2F. Finally, for 50 cycles, while the hard mask was still partially present although showing clear signs of degradation (Figure 2G), the cross section demonstrated the uncontrolled formation of pillars, possibly resulting from the localized collapse of the pore walls required to separate the pores, as well as the creation of nanograss structures (Figure 2H) commonly occurring in processes involving highly directional plasma etching of silicon.^43^ The observed limitations concerning the process reliability at increasing nanopore depths are expected to be associated with the increasing reduction in gas exchange, which leads to a lack of fluorocarbon needed for the deposition of the passivation layer as well as insufficient removal and subsequent redeposition of byproducts.

**Figure 3:**
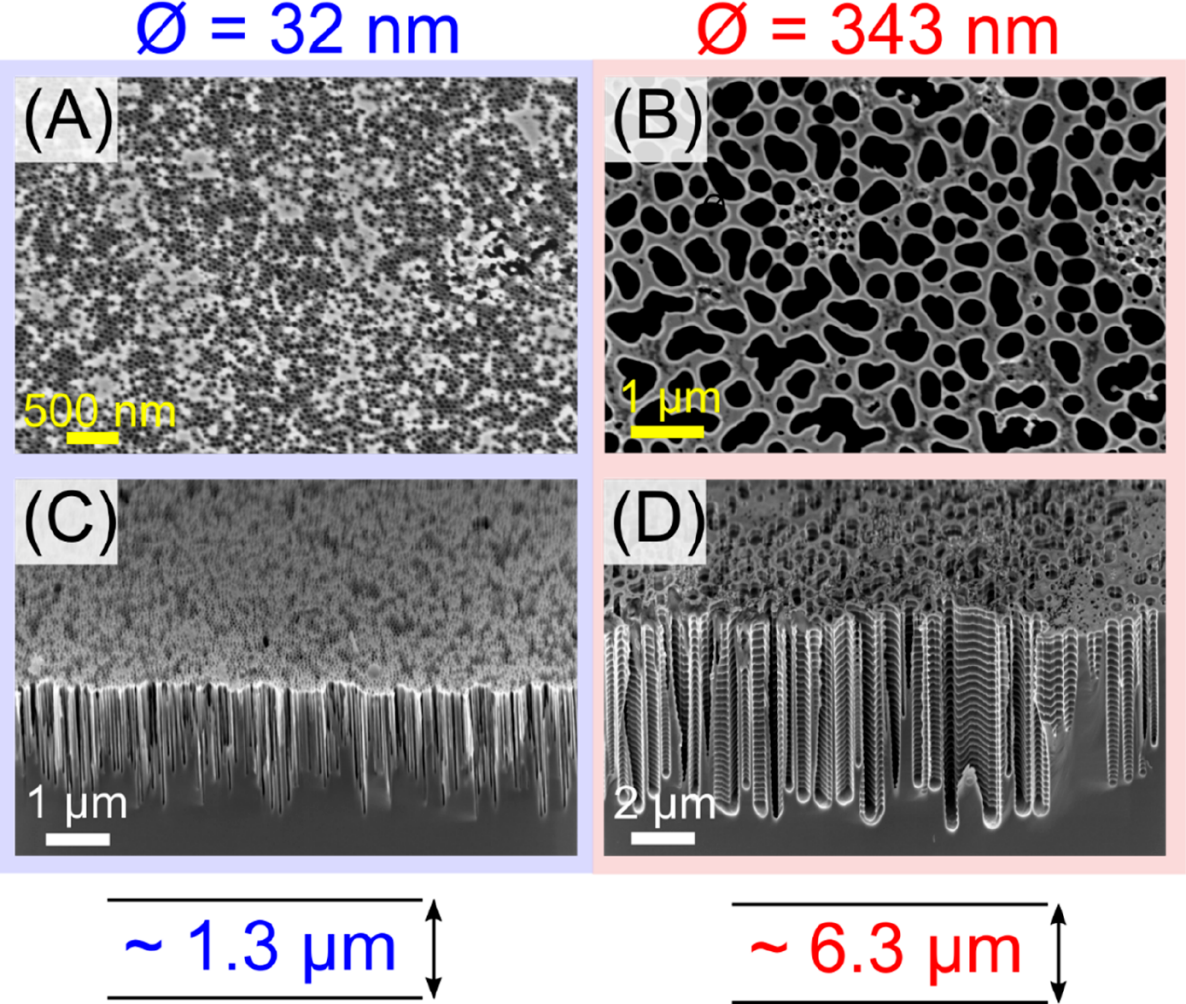
For 343 nm masks, the etch depth increases, but the etch quality is reduced. The figures show top down and cross section SEM micrographs of the etched silicon substrates with varying diameters (Ø): (A,C) 32 nm and (B,D) 343 nm.

To assess the effect of pore sizes on the etching performance and device quality, samples with different pore diameters, i.e., 32 and 343 nm (Figure 3), were etched using the same protocol and equal number of cycles (30). For 343 nm pores, a depth of 6.25 ± 0.61 μm was obtained, which is a 5-fold increase in etch depth compared to the 32 nm pores, however, simultaneously a drop in aspect ratio to 18. This decrease in the aspect ratio can be attributed to the highly modified proportion of chemical etching in our newly developed etching process cycle, which, while ideal for narrow features, is not optimized for wider structures, as can also be seen by the formation of scallops along the walls of the pores (Figure 3D). Nevertheless, as etch performance is directly linked to constraints provided by the penetration of the ions during the etching and the transport of the reactants, products, and byproducts, the simultaneous optimization for multiple features with strongly varying dimensions is commonly acknowledged as especially challenging, as often prominently highlighted through the presence of RIE lag or etch lag also mentioned above.^49^ Additionally, only minor changes in pore sizes and pore–pore distances throughout the patterning process, from the BCP thin film to the final etched surface, were detected (SI Table S1 and Figure S4).

### Nanoscale Porous Silicon Substrates Demonstrate Suitability for Potential Applications in Light Absorption and Thermal Management

**Figure 4:**
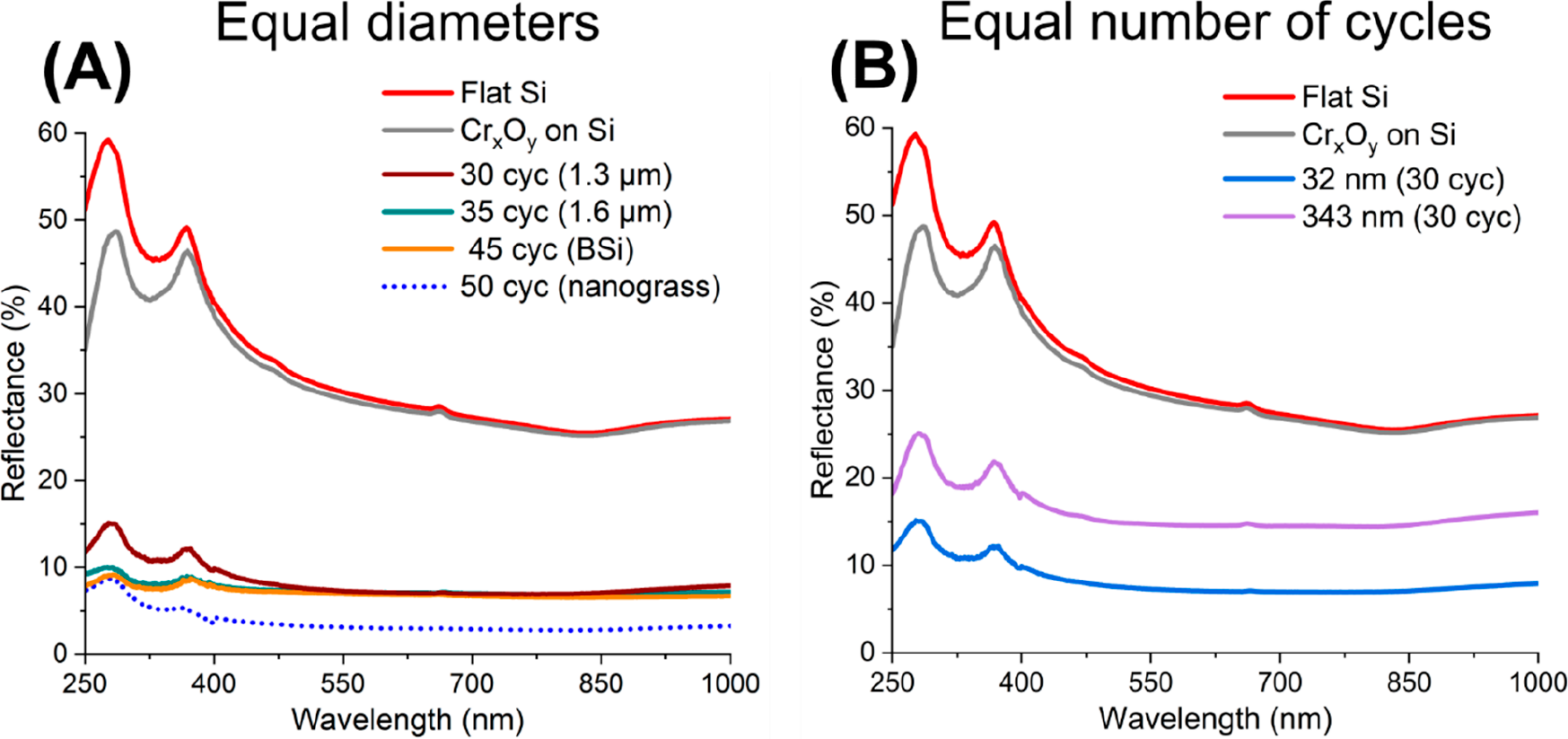
Performed etching significantly improves absorption for both pore diameters. The figures report reflectance measurements in the range of 250 to 1000 nm for (A) different etch cycles with 32 diameter pores and (B) different pore sizes.

As a part of the device’s primary interaction with the environment, surface reflectance and absorption play crucial roles in the optical performance of microdevices and their subsequent application. Hence, standard reflectance measurements at a normal incidence angle were performed to quantify the optical properties and evaluate the suitability of the porous substrates for antireflection coatings. Overall, the spectra of the devices with varying depths demonstrated similar qualitative behaviors with a decrease in reflectance for increasing numbers of etch cycles. Further, increasing depths led to an attenuation of the interband transition peaks,^50^ as observable at 272 and 364 nm (Figure 4A). For reference, the flat surface of a polished silicon substrate, i.e., without mask fabrication or subsequent etching, presented an average reflection value across the visible range (380–700 nm) of 26%. Additionally, to analyze the effect of any Cr oxide remaining after the etching process, the reflectance of a Si substrate covered with a Cr oxide mask with 32 nm pores was measured, and an average reflectance value of 25%, similar to the one obtained for a planar silicon substrate, was detected, which confirmed that the mask itself did not contribute significantly to the change in optical properties. Then, the variation of etch cycles and their effect on the reflectance were assessed in further detail. Both, the samples with 30 and 35 cycles, had an average reflectance value of approximately 7% in the visible range. However, in the UV range, the intensity of the interband transition peaks for the 35-cycle sample were significantly reduced when compared to the 30-cycle one. Overall, both numbers of cycles presented similar spectra, which can likely be accounted for by both processes leading to a nanostructure depth at which their intensity along the spectrum reached a stable minimum.^51^ Additionally, for the 45-cycle sample, although an early collapse of the walls separating the pores was observed in the SEM analysis, the reflection value was found to be very similar to 35, further suggesting a plateau in reflectance for samples with diameters of 32 nm around this depth. Finally, the 50-cycle sample demonstrated an exceptionally low average reflectance value in the visible range of only 2.7%. However, this extra reduction can likely be assigned to the formation of the nanograss-like black silicon (BSi) features described above, which can create graded refractive index profiles at the interface between the air and the silicon and, by that, improve the material’s light-absorbing characteristics.^52^ Nevertheless, given the reduced reliability in the formation of BSi structures during the use of our protocol, the fabrication and application of stable devices, such as those achieved with 35 etch cycles, would be suggested. To summarize, it is worth highlighting that our samples demonstrate a high absorption of light in the ultraviolet, visible, and infrared A ranges and do not rely on antireflection coatings, which makes them useful for various applications in need of light-absorbing surfaces.

In a next step, we analyzed the effect of the pore width onto the derived reflectance. Figure 4B shows a reflectance comparison for the two different pore diameters. The 343 nm sample demonstrated an average reflectance value of 14% in the visible range, which is substantially greater than the 7% detected for the 32 nm sample. This difference in performance suggests that the optical behavior of our samples is related to the moth-eye effect, in which subwavelength features form a continuous refractive index between the air and the surface that reduces light reflections.^53,54^ Accordingly, it could be assumed that the 32 nm sample allows for a higher capacity in absorbing light when compared to the 343 nm sample due to its smaller dimension as well as its higher feature density. Alternatively or simultaneously, the increase in reflectance could also be caused by an increase in diffuse light scattering related to the increasing pore size.^55^

**Figure 5:**
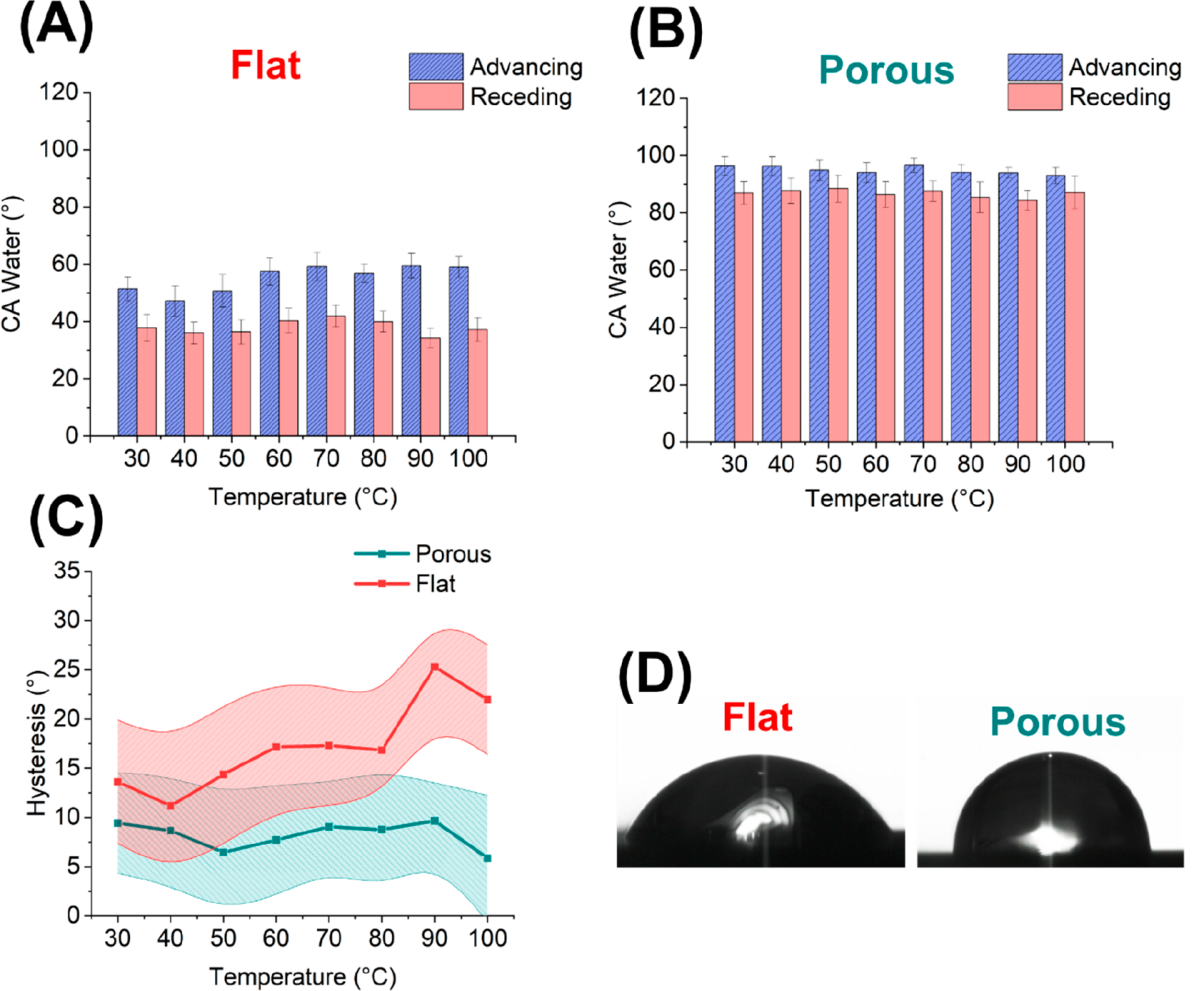
Surface modification improves device cooling performance and hydrophobicity. The figures present the advancing and receding water contact angles for (A) flat and (B) porous silicon substrates with 32 nm pore diameter and 35 etch cycles (*n* = 10). (C) Contact angle hysteresis (shaded areas = 95% confidence interval). (D) Droplet images at 30 °C.

To study the potential application of our structures in thermal management, water contact angles (WCAs) were measured at different temperatures for flat silicon and processed (35 cycles) silicon substrates based on 32 nm pores. For the flat silicon substrate, the WCA showed a hydrophilic character (Figure 5A) with an average advancing value of ∼55° and a receding angle of ∼38°. A slight increase in the advancing contact angle was observed by increasing the temperature to approximately 60 °C. After the fabrication of the porous patterns, the WCA displayed an increase in the hydrophobic character of the surface (Figure 5B), reaching values of ∼95° for the advancing and ∼87° for the receding angles. This behavior can be explained by the Cassie–Baxter wettability regime, in which nanostructured surfaces are able to trap air, leading to a reduction in the surface wetted by the liquid.^56,57^ Accordingly, the Cassie–Baxter equation can be simplified as follows when air pockets are present on a rough single-component surface:^58^
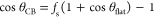
1where θ_CB_ is the contact angle for the porous surface, *f*_s_ is the fraction of the water/solid contact surface area, and θ_flat_ is the contact angle of the flat/smooth surface.

Considering that the average static contact angle of the flat surface is 55° and *f*_s_ is ∼65% (pore surface area: ∼35% (for 32 nm/35 cycles) — based on SEM micrographs), the contact angle θ_CB_ is about 89°, which agrees well with the experimental data.

Subsequently, the hystereses of the advancing and receding contact angles were calculated (|θ_advancing_ – θ_receding_|) (Figure 5C). The hysteresis arises from the dissimilarity in the interaction between the liquid and solid surfaces at the contact line during the advancement and recession of the liquid.^59^ Compared to the flat surface, the etched sample, on average, demonstrated lower values, meaning that the advancing and receding WCA were more alike, which led to improved balancing of tensions at the liquid–solid interfaces. It has previously been shown that a lower hysteresis also permits improved heat transfer,^60^ with further reports highlighting that the critical heat flux is reduced when the contact angle hysteresis increases.^61^ Moreover, lower CA values have been found to decrease the boiling heat efficiency in thermal systems.^62^ Finally, the presence of the pores in the substrate can enhance the heat transfer via the increased surface area in parallel with capillary wicking, while pore uniformity, as demonstrated for 32 nm masks and 35 etching cycles, has also been highlighted to play a key role in liquid evaporation.^63,64^ Hence, while further testing is required, compared to flat substrates, our processed material is expected to display a significantly improved performance when applied for thermal management and heating/cooling systems. Nevertheless, prior to final applications or implementations, it is generally suggested that the relationship between the precise surface chemistry and the device performance should be studied in further detail to ensure long-term stability and reliability.

## Conclusion

To conclude, the optimized DRIE process in combination with the advanced BCP-templated mask fabrication on silicon substrates presents a promising method to produce deep porous structures at the nanoscale, which is further amplified by the low-cost strategy and versatility applied during its production. Additionally, the developed procedures and corresponding devices have proven valuable for different applications, including tasks relying on low reflectance values, such as required in light sensing or absorption, as well as for thermal management via nanostructured substrates, as relevant in electronics, and are expected to further support advancements in miniaturization and membrane manufacturing.

## Supporting Information Available

AFM images for the determination of the optimal Cr precursor concentration, schematic of the etching process highlighting each step, (S)TEM/EDX of an etched sample, and pore size and pore-pore distance statistical analysis (PDF)
