# Supplementary material for: High Aspect Ratio Nanoscale Pores through BCP-Based Metal Oxide Masks and Advanced Dry Etching
Source: ACS Appl Mater Interfaces. 2023 Oct 20;15(50):57960–9. doi: 10.1021/acsami.3c09863 (PMC10739579; doi:10.1021/acsami.3c09863)
Supplement: Supplementary file 1 — am3c09863_si_001.pdf [file am3c09863_si_001.pdf]

SUPPORTING INFORMATION

High Aspect Ratio Nanoscale Pores through BCP-Based Metal Oxide Masks and Advanced Dry Etching

Aislan Esmeraldo Paiva,[1*] Michael S. Gerlt,[2, 3*] Nino F. Läubli,[4] Nadezda Prochukhan,[1] Jhonattan Frank Baez Vasquez,[1] Gabriele S. Kaminski Schierle,[4] Michael A. Morris.[1*]

*esmerala@tcd.ie *michael.gerlt@bme.lth.se *morrism2@tcd.ie

AUTHOR ADDRESS

1) AMBER Research Centre/School of Chemistry, Trinity College Dublin, Dublin, D02CP49, Ireland.

2) Department of Biomedical Engineering, Lund University, Lund, 223 63, Sweden.

3) Department of Mechanical and Process Engineering, ETH Zürich, Zürich, 8092, Switzerland.

4) Department of Chemical Engineering and Biotechnology, University of Cambridge, Cambridge, CB3 0AS, UK.

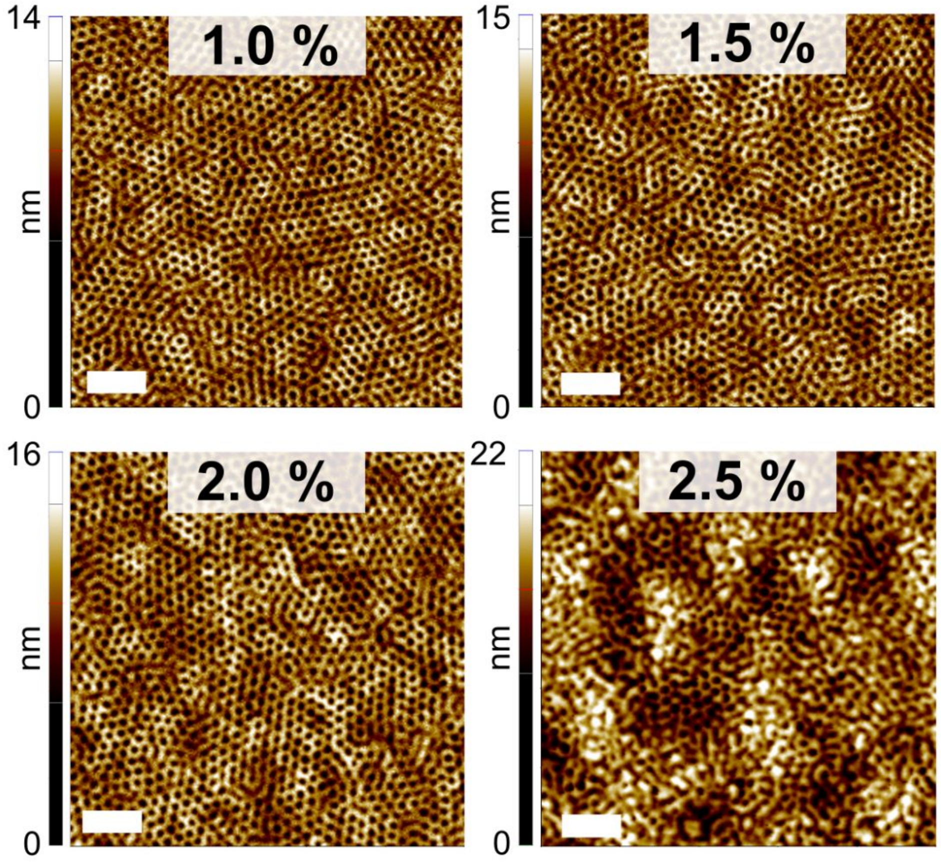

Figure S1. Variation in chromium precursor concentration (% wt.) and its effect on morphology for BCP1. All scale bars are 300 nm.

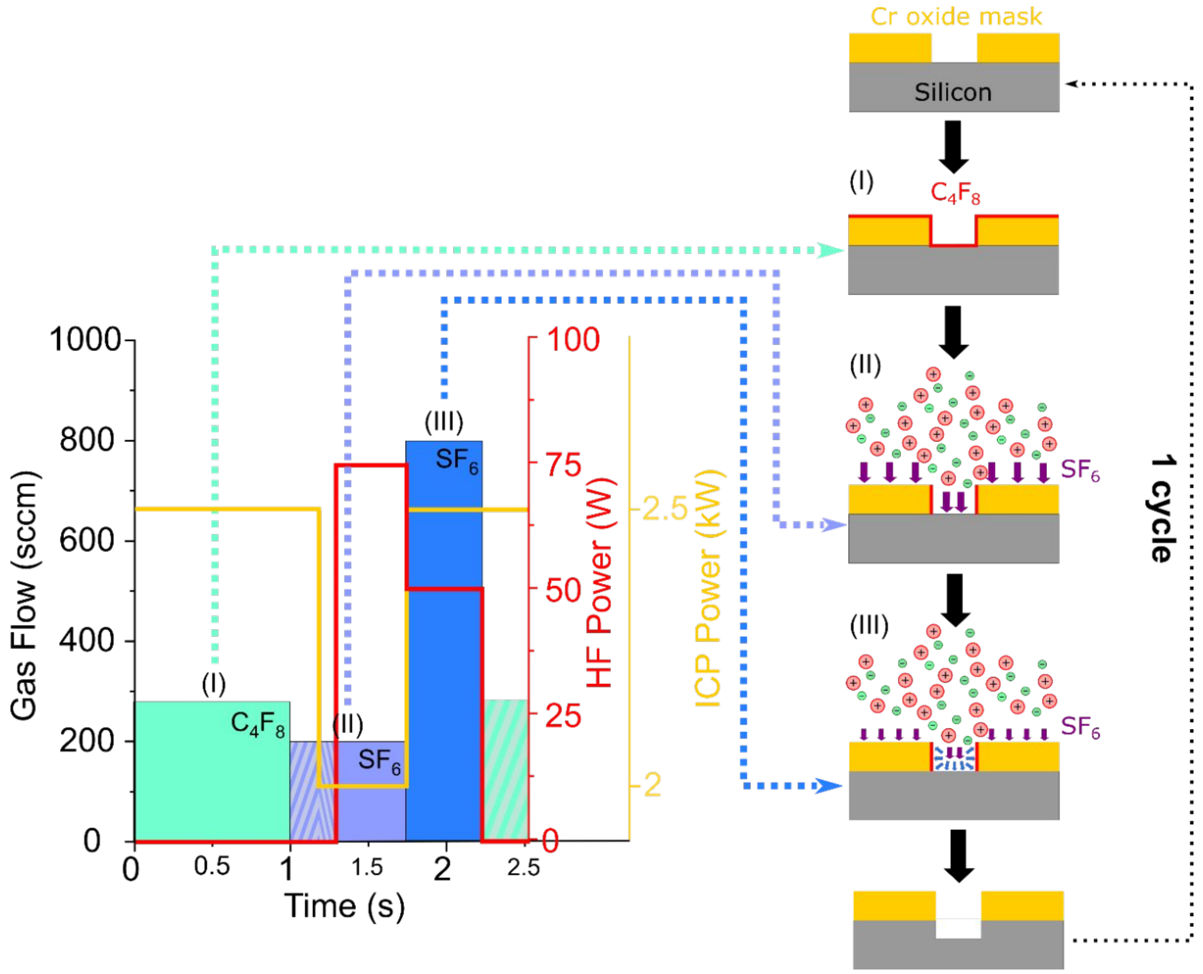

Figure S2. Schematic of the etching cycle with the three main steps for passivation (I), anisotropic break through etching (II), and isotropic Si etching (III), as well as the intermediate steps (grey boxes) used to prepare the chamber and sample for the subsequent procedure *i.e.*, through the exchange of the gas environment, the adjustment of the ICP power, or the phasing out of the passivation.

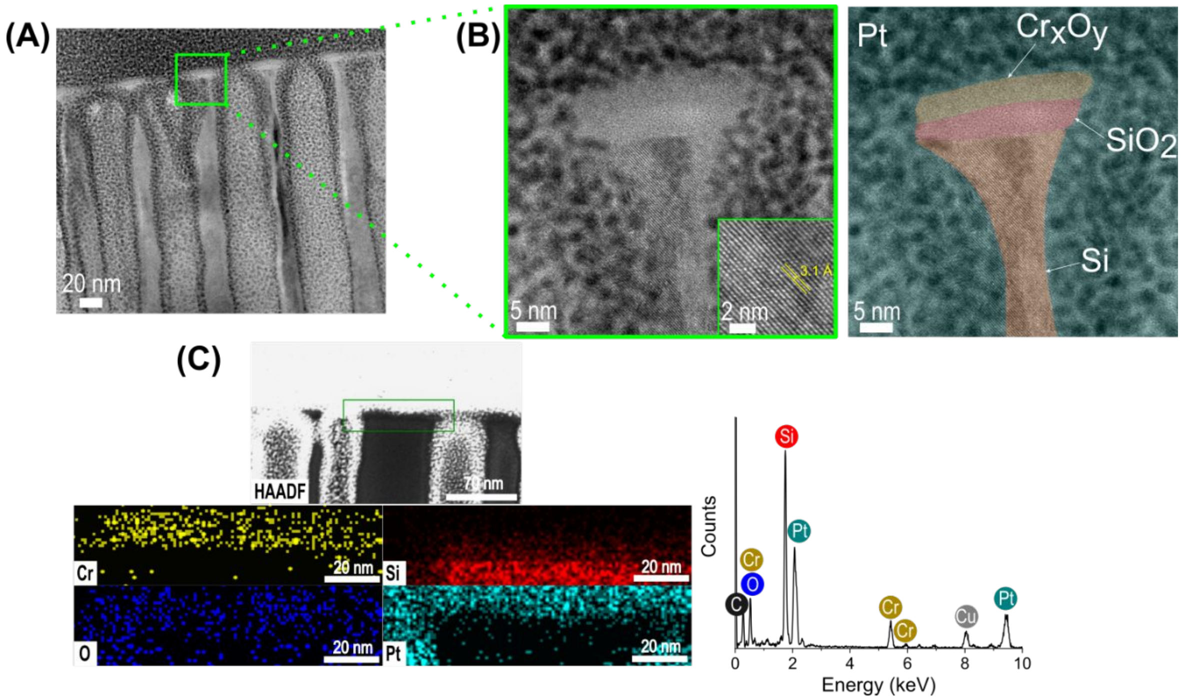

Figure S3. (A) TEM image of the porous silicon substrate (35 nm diameter pores). (B) High-resolution TEM of a pore wall and corresponding colour-coded image highlighting the different materials (Pt from capping protective layer, Cr_xO_y hard mask, SiO_2 native oxide layer and Si). The inset shows the lattice fringes of the Si structure ((111) plane - FCC). (C) STEM/EDX elemental mapping.

**Pore size (nm)**	**BCP 1**	**Cr oxide 1**	**Etched Si 1**
	34 ± 3	32 ± 4	37 ± 9
	**BCP 2**	**Cr oxide 2**	**Etched Si 2**
	400 ± 120	343 ± 85	366 ± 74
**Pore- pore distance**	**BCP 1**	**Cr oxide 1**	**Etched Si 1**
	60 ± 4	60 ± 5	67 ± 11
	**BCP 2**	**Cr oxide 2**	**Etched Si 2**
	541 ± 115	460 ± 86	480 ± 60

Table S1. Pore sizes and pore-pore distances quantified *via* AFM and SEM. The etched samples correspond to 30 cycles.

For pore sizes, a change in size of ~ 8 % was detected from the BCP templates to the final material. Regarding the pore-pore distance, ~ 11 % variation in value was observed.

An additional analysis was carried out for the pore-pore distances based on power spectral density (PSD). The PSD spectra were obtained via the AFM and SEM images (**Figure S4**). For BCP 1, pore-pore distances were found to be 50 nm, while for the respective Cr oxide mask (Cr oxide 1), 56 nm. For BCP 2, a pore-pore distance of 588 nm was detected, while it was 500 nm for the Cr oxide 2. Both results show minor changes in the sizes. For the etched surfaces, the PSD analysis did not show any clear peak which could be assigned to the pore-pore distance.

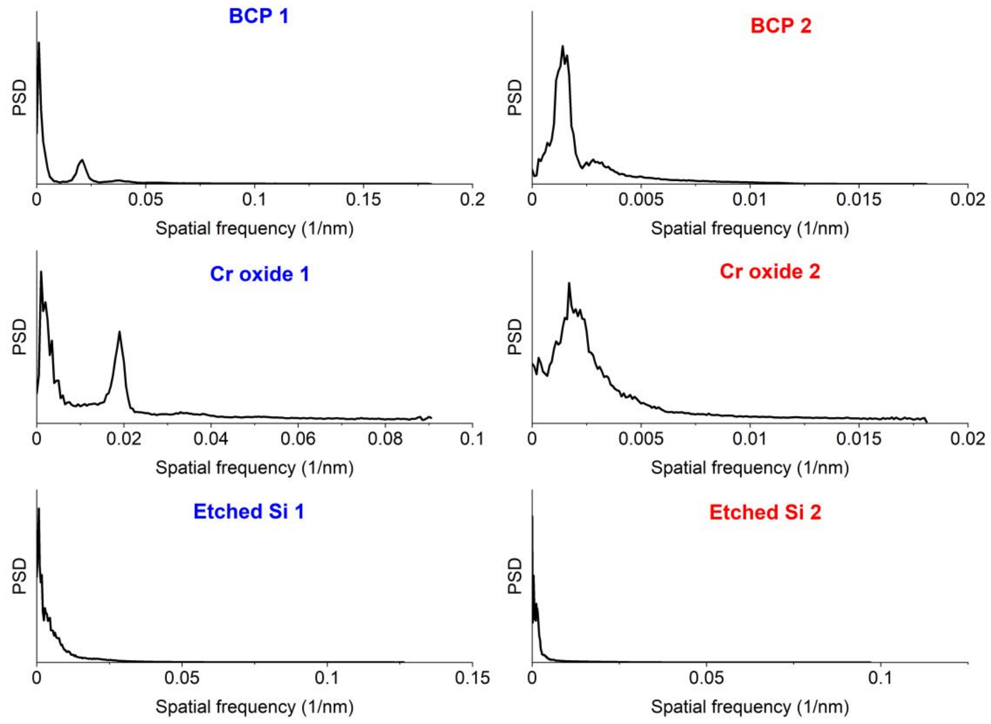

Figure S4: PSD spectra for the different BCP templates, Cr oxide masks, and etched silicon substrates. The spectra were calculated *via* the FFT of the AFM and SEM images.
